# A case of early onset adenocarcinoma associated with colorectal polyposis with an unknown germline mutation

**DOI:** 10.1186/s40792-022-01518-2

**Published:** 2022-08-25

**Authors:** Masahiro Zenitani, Hidehito Inagaki, Hiroki Kurahashi, Takaharu Oue

**Affiliations:** 1 Department of Pediatric Surgery, Hyogo College of Medicine, 1-1, Mukogawa, Hyogo 663-8501 Nishinomiya, Japan; 2 Division of Molecular Genetics, Institute for Comprehensive Medical Science, Fujita Health University, Aichi, Japan

**Keywords:** Polyposis, Adenocarcinoma, Children, Pediatric, Mutation

## Abstract

**Background:**

Typically, in cases of adenomatous polyposis, colorectal cancer develops in the third or fourth decade of life. We report the case of a female patient with colorectal polyposis who developed adenocarcinoma at 8 years of age.

**Case presentation:**

An 8-year-old girl was admitted with a 4-year history of occasional bloody stools. Colonoscopy revealed colon polyposis and histopathological assessment confirmed a well-differentiated adenocarcinoma in the adenomatous polyps, so laparoscopy-assisted proctocolectomy was performed in the lithotomy position by a simultaneous abdominal and anal approach. To completely resect the rectal mucosa, excision was commenced just distal to the dentate line. After the mucosal resection up to the peritoneal reflection level, an inverted muscular cuff was cut circumferentially, and the terminal ileum was pulled through the muscular cuff and anastomosed to the anal canal. Histopathology revealed multiple adenomatous polyps and scattered well-differentiated tubular adenocarcinomas (tub1) in the adenomatous polyps and the non-polypoid mucosal lesions. Because complete resection was achieved, additional adjuvant chemotherapy was not administered. Polymerase chain reaction (PCR)-direct sequencing of the entire coding region and the exon–intron junctions, and real-time PCR of DNA extracted from blood cells, revealed no mutations of either *APC* or *MUTYH*. No deletions, duplications, translocations or inversions of *APC*, *MUTYH* and *GREM1* genes were found using multiplex ligation-dependent probe amplification (MLPA) and G-banding analysis. Multi-gene panels sequencing for polyposis syndromes or hereditary colorectal cancers, and trio-whole exome sequencing was conducted. However, no candidate pathogenic variants of genes were detected in de novo dominant or autosomal recessive model. Somatic mutation of *APC* was not detected in 4 polyps by loss of heterozygosity analysis at a single nucleotide polymorphism in intron 14. The patient has remained disease-free for 5 years. Currently, the patient is on loperamide and passes stool 5 times/day without any soiling.

**Conclusions:**

The genetic analysis suggests that she may have a germline mutation at unscreened region of these genes or in unidentified FAP gene. The patient will be carefully followed up for residual rectal carcinoma and for the development of other cancers.

## Background

Familial adenomatous polyposis (FAP) is a rare hereditary colorectal disease, occurring in approximately 1 in 17,400 births in Japan [1]. Most patients with FAP have a mutation in the adenomatous polyposis coli (*APC*) gene on chromosome 5q22. The average age of classic FAP onset is 16 years, and adenocarcinoma develops at a mean age of 45 years in these patients [2]. No cases of colorectal cancer at or before the age of 10 years, and only two incidental cases between the ages of 11 and 15 years were found in the European registry of FAP patients with colorectal cancer (0.19%, 2/1073 of total patients) [3]. To the best of our knowledge, no cases of colorectal cancer in FAP patients younger than 10 years of age have been reported. When evaluating patients with early onset of multiple adenomatous polyps in the absence of proven *APC* germline mutations, the differential diagnosis needs to include attenuated-FAP, mutY homologue (*MUTYH*)-associated polyposis and constitutional mismatch repair deficiency (CMMRD) syndrome [4, 5]. We report the case of a female patient with colorectal polyposis who developed adenocarcinoma at 8 years of age. We demonstrate her clinical course, particularly focusing on our surgical treatment and genetic analysis.

## Case presentation

**Fig. 1:**
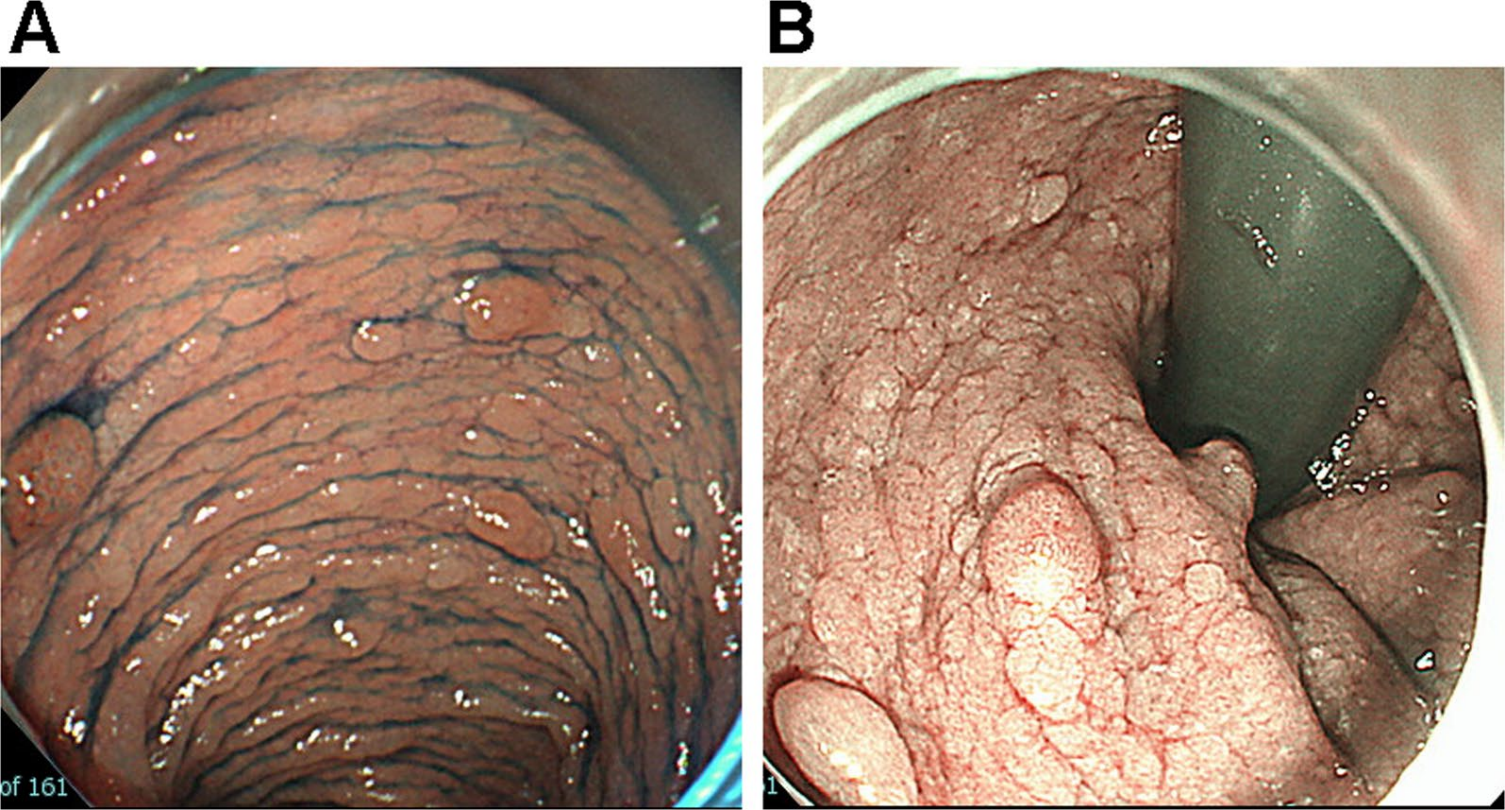
Endoscopic view of profuse polyposis at the colon (**A)** and rectum (**B**)

An 8-year-old girl was admitted with a 4-year history of occasional bloody stools. Colonoscopy revealed profuse colorectal polyposis (Fig. 1). Pathological assessment confirmed well-differentiated adenocarcinoma in adenoma in 4/14 resected adenomatous polyps. The histological diagnosis was intramucosal adenocarcinoma (pTis) in adenoma. Upper gastrointestinal endoscopy and contrast computed tomography (CT) showed no extra-colorectal lesions associated with FAP and no metastatic lesions. There was no family history of colorectal polyposis. Colonoscopies confirmed that neither her parents nor her younger brother had colorectal polyposis.

### Surgical procedure

Laparoscopy-assisted proctocolectomy was performed in the lithotomy position by a simultaneous abdominal and anal approach.

Abdominal approach: An umbilical incision was made, and mesentery from the terminal ileum to the sigmoid colon was dissected just proximal to the marginal vessels (D1 lymph node resection). The mesentery from the sigmoid colon to the rectum below the peritoneal reflection was laparoscopically dissected. After the transection of sigmoid colon with a linear cutting stapler, the colon from cecum to sigmoid was extracted from the umbilical incision.

**Fig. 2:**
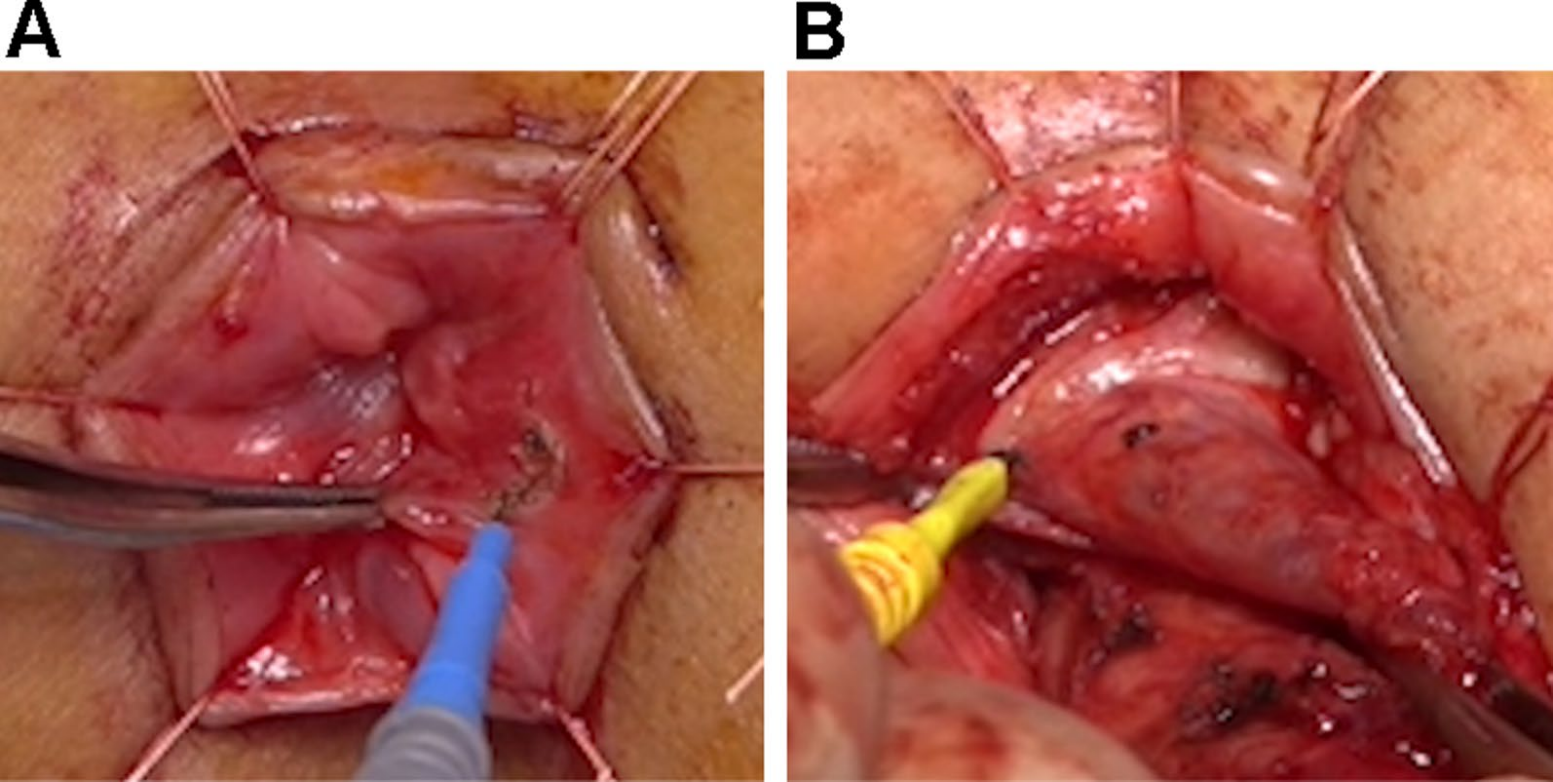
Surgical procedure for complete resection of rectal mucosa. **A** Excision was commenced just distal to the dentate line. **B** Transanal rectal mucosectomy was performed as in a Soave procedure for Hirschsprung’s disease

Anal approach: To completely resect the rectal mucosa, excision was commenced just distal to the dentate line (Fig. 2A). Endorectal resection of the mucosa and submucosa was performed as in a Soave endorectal pull-through procedure for Hirschsprung’s disease (Fig. 2B). After the mucosal resection up to the peritoneal reflection level, an inverted muscular cuff was cut circumferentially. After the remaining sigmoid colon and rectum was extracted from the anus, the terminal ileum was pulled through the muscular cuff and anastomosed to the anal canal. Operative time was 264 min, blood loss was 55 mL, and there were no operative complications.

### Histopathological findings

**Fig. 3:**
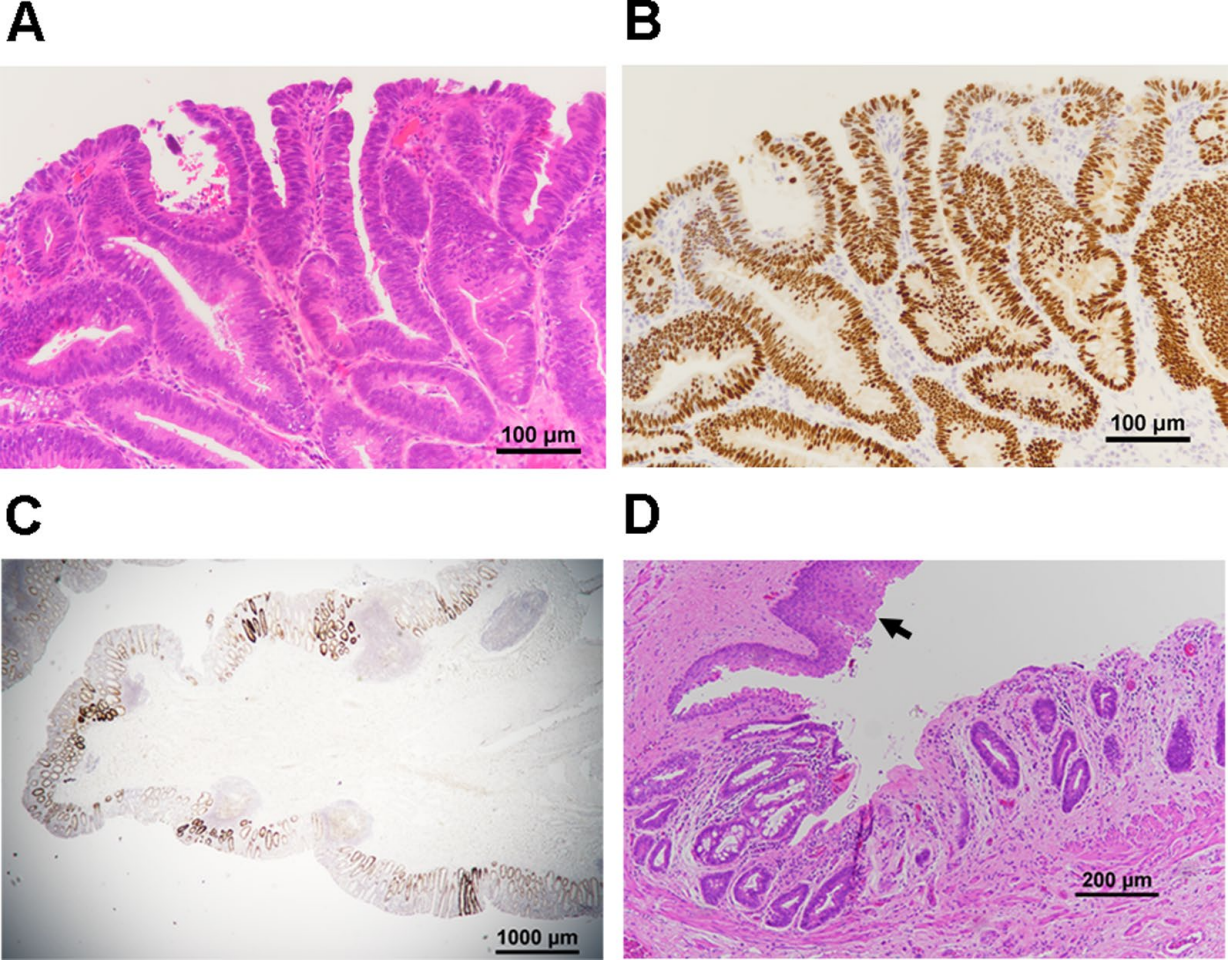
Histopathological findings of resected colorectum.. Staining of hematoxylin and eosin **A** and p53 **B** in a resected specimen with a magnification of × 200 showed well-differentiated tubular adenocarcinomas. **C** Strongly positive p53 staining which was expressed on highly atypical or adenocarcinoma components was also scattered in the non-polypoid mucosal lesions. **D** Distal resection margin was covered with stratified squamous epithelium (arrow)

Tubular adenomas were found in the entire colon and well-differentiated tubular adenocarcinomas (tub1) (Fig. 3A, B) were detected in four sections of the rectum. All the carcinomas were classified as pTis (M) as well as in the polypectomy specimen. Histopathology revealed multiple adenomatous polyps and scattered highly atypical or adenocarcinoma components in the adenomatous polyps and the non-polypoid mucosal lesions (Fig. 3C). Stratified squamous epithelium covered the entire circumference of the distal resection margin (Fig. 3D). No metastases were observed in the peri-colorectal lymph nodes.

### Genetic analysis

After genetic counseling, the patient and her parents underwent genetic testing. Polymerase chain reaction (PCR)-direct sequencing of the entire coding region and the exon–intron junctions, and real-time PCR of DNA extracted from blood cells, revealed no mutations of either *APC* or *MUTYH*. No deletions or duplications of *APC*, *MUTYH* and *GREM1* genes were found using multiplex ligation-dependent probe amplification (MLPA) (SALSA MLPA probemix P043-E1; MRC, Amsterdam, The Netherlands). No translocation or inversion of these genes was confirmed by G-banding analysis. Although next-generation target resequencing using a panel of 94 cancer related genes (TruSight cancer panel, Illumina Japan, Tokyo, Japan) was performed to explore the hereditary component associated with polyposis syndromes including *APC*, *MUTYH*, *BMPRA1*, *SMAD4* and *PTEN* genes, no mutations were detected. Multi-gene panel sequencing using MHCRCv3 to detect hereditary colorectal cancer-related genes, including *MLH1*, *PMS2*, *MSH2*, *MSH6*, *EPCAM*, *MSH3*, *MBD4*, *APC*, *MUTYH*, *NTHL1*, *POLD1*, *POLE*, and *TP53* was conducted, using the method described by Makabe et al. [6]. Trio-whole exome sequencing was performed using SureSelect Clinical Research Exome (Agilent Technologies Japan, Ltd.). However, no candidate pathogenic variants were detected in de novo dominant or autosomal recessive model, nor pathogenic variants on highly scored-polyposis related genes in GeneCards website. Somatic mutation of *APC* was not detected in 4 polyps by loss of heterozygosity analysis at a single nucleotide polymorphism in intron 14 (rs1217729675).

### Postoperative clinical course

Complete resection was achieved, so no additional adjuvant chemotherapy was administered. Surveillance endoscopy and contrast CT were performed yearly for detection of extra-colorectal lesions and residual rectal carcinoma. The patient has remained disease-free for 5 years. Currently, the patient is on loperamide and passes stool 5 times/day without any soiling.

## Discussion

In the present patient, genetic analysis detected no germline mutations of FAP (*APC*), MUTYH-associated polyposis (*MUTYH*), polymerase proofreading-associated polyposis (*POLD1* and *POLE*), CMMRD syndrome (*MLH1*, *PMS2*, *MSH2*, *MSH6* and *EPCAM*), juvenile polyposis syndrome (*BMPRA1, SMAD4*), Cowden disease (*PTEN*), hereditary mixed polyposis syndrome (*GREM1*), NTHL-1 associated polyposis (*NTHL1*), MSH3-associated polyposis (*MSH3*) and Li–Fraumeni syndrome (*TP53*). This suggests that she may have a germline mutation at unscreened region of these genes or in unidentified FAP gene.

In adult patients, ileal pouch–anal anastomosis (IPAA) is generally performed after proctocolectomy; however, outcomes remain controversial for pediatric patients undergoing straight ileoanal anastomosis (SIAA) vs IPAA [7]. Seetharamaiah et al. carried out a multicenter analysis of stooling scores from 250 children after proctocolectomy with either SIAA or IPAA and found that stooling scores became similar at 2 years after either operation. Thus, continence was excellent regardless of the technique [7]. In our patient, complete resection of rectal mucosa was achieved by beginning with the mucosal resection of the rectum distal to the dentate line, resulting in no recurrence for 5 years postoperatively. Moreover, preserving the muscular layer of the rectum might contribute to tolerable continence. Therefore, this surgical procedure is considered favorable.

There is a risk of developing adenoma or carcinoma in the ileal pouch among FAP patients who have undergone proctocolectomy and IPAA [8, 9]. Tajika et al. reviewed 25 reports regarding these risks and described that the risk of adenoma appears to be 7–16% after 5 years, 35–42% after 10 years, and 75% 15 years after the operation. They also described that the median duration between the operation and the diagnosis of pouch carcinoma in 21 cases was 10 years (range, 3–20 years) [8]. Friederich et al. reported that among 254 patients with FAP who underwent proctocolectomy and IPAA selected from the Dutch polyposis registry, a cumulative risk of developing a pouch carcinoma at 10-year follow-up was 1% [9]. In the present case, residual rectal carcinoma would develop more rapidly compared to classic FAP. Motivated by these findings, we recommend yearly endoscopic surveillance for at least 10 years postoperatively and possibly throughout her adult life.

## Conclusions

The present case highlights the rare, aggressive nature of colorectal polyposis and adenocarcinoma with an unknown germline mutation. We also demonstrate a feasible surgical procedure that might aid in the prevention of recurrence for 5 years postoperatively and result in tolerable continence. The patient will be carefully followed up for residual rectal carcinoma and for the development of other cancers due to the unknown nature of her mutation and the possibility of having genes that predispose her to other types of cancer.

## Data Availability

Data sharing is not applicable to this article as no data sets were generated or analyzed.

## Abbreviations

FAP: Familial adenomatous polyposis
APC: Adenomatous polyposis coli
MUTYH: MutY homologue
CMMRD: Constitutional mismatch repair deficiency
CT: Computed tomography
PCR: Polymerase chain reaction
MLPA: Multiplex ligation-dependent probe amplification
IPAA: Ileal pouch–anal anastomosis
SIAA: Straight ileoanal anastomosis
